# Biomechanical evaluation and finite element analysis of coracoclavicular ligament reconstruction using a modified double-pulley technique

**DOI:** 10.3389/fbioe.2026.1910380

**Published:** 2026-09-28

**Authors:** Qi Zhang, Shunlin Zhang, Song Chen, Yongyue Cheng, Wuxiang Wang, Jinhui Liu, Hongbin Yang

**Affiliations:** 1 Department of Orthopedics and Joint Surgery, the Affiliated Hospital of Southwest Medical University, Luzhou, Sichuan, China; 2 Department of Orthopedics, Luzhou People’s Hospital, Luzhou, Sichuan, China; 3 Department of Orthopedics, the First Hospital of Zhaotong City, Zhaotong, Yunnan, China; 4 Sichuan Provincial Engineering Laboratory for Research and Application Technology of Orthopedic Implant Devices, Luzhou, Sichuan, China

**Keywords:** acromioclavicular joint dislocation, biomechanics, coracoclavicular ligament reconstruction, finite element analysis, modified double-pulley technique

## Abstract

**Introduction:**

Conventional point-to-point (PTP) suture-anchor fixation for coracoclavicular ligament reconstruction may lead to uneven load sharing and stress concentration. This study evaluated the biomechanical performance of a modified double-pulley (DP) technique and compared it with the conventional PTP method using synthetic bone testing and finite element analysis.

**Methods:**

Ten clavicle-coracoid polyurethane model pairs were randomly assigned to the modified DP group or PTP group, with five pairs in each group. All constructs underwent cyclic loading followed by load-to-failure testing. Initial displacement, final displacement, displacement change, ultimate failure load, and failure mode were recorded. Finite element models were also established to compare stress distribution and distal clavicle displacement between the two configurations under identical boundary and loading conditions.

**Results:**

The modified DP group showed significantly lower initial displacement, final displacement, and displacement change than the PTP group during cyclic loading. The ultimate failure load was significantly higher in the modified DP group than in the PTP group (367.07 ± 17.24 N vs. 285.70 ± 38.48 **N**, p < 0.01). Failure occurred through bilateral anchor pullout in the modified DP group but unilateral anchor pullout in the PTP group. Under the specified mesh and modeling conditions, the modified DP model showed lower computed peak stresses in the suture (196.01 MPa vs. 287.72 MPa), anchor (196.01 MPa vs. 274.82 MPa), and clavicle (26.54 MPa vs. 32.99 MPa), as well as lower distal clavicular displacement (0.23552 mm vs. 0.24565 mm), than the PTP model.

**Conclusion:**

The modified DP technique provided greater initial fixation strength and lower cyclic displacement than the conventional PTP method under the present synthetic-model testing conditions. The finite element findings provided exploratory, model-dependent mechanistic support for the observed biomechanical differences.

## 1 Introduction

Acromioclavicular (AC) joint dislocations are common shoulder injuries typically resulting from direct or indirect high-energy trauma to the acromion. These injuries account for approximately 2%–16% of all joint dislocations and are particularly prevalent among athletes and adolescents (Babhulkar and Pawaskar, 2014; Simovitch et al., 2009). Surgical intervention is generally indicated for high-grade Rockwood types IV-VI and type III injuries in patients with high functional demands (Bradley and Elkousy, 2003; Cote et al., 2010). The primary goal of surgical management is to restore the anatomical alignment between the clavicle and the coracoid process, thereby ensuring vertical stability of the AC joint and facilitating healing of the coracoclavicular (CC) ligaments (Bigoni et al., 2019; Frank et al., 2019). Rigid fixation techniques such as Kirschner wires, tension band wiring, titanium cables, CC screws, and hook plates have been widely used to stabilize the CC distance (Leidel et al., 2009; Stucken and Cohen, 2015; Sundaram et al., 1992). However, these techniques are associated with complications including hardware breakage, loosening, migration, osteolysis, and even coracoid fractures (Ko and Lee, 2019; Łazarski et al., 2019). Consequently, attention has shifted toward flexible fixation methods that better mimic the biomechanical properties of native ligaments and accommodate micromotions during healing (Oki et al., 2012).

Suture anchors have emerged as a promising option for CC ligament reconstruction by establishing a stable suture loop between the clavicle and coracoid, promoting scar formation and functional recovery. Most existing techniques use a point-to-point (PTP) configuration with two independent anchors, which lacks mechanical synergy. Discrepancy in tension between the anchors can result in load concentration on a single anchor, increasing the risk of anchor pullout and fixation failure (Liu et al., 2020). Double-pulley (DP) technique, originally developed by Arrigoni et al. for rotator cuff repair (Arrigoni et al., 2007), interconnects two anchors using crossing sutures to form a downward compressive force, effectively redistributing stress and enhancing pullout resistance. This approach has demonstrated improved fixation and stability in Bankart lesions and other orthopedic applications (Zhang and Jiang, 2011), though its biomechanical properties in CC ligament reconstruction remain underexplored.

Finite element analysis (FEA) is a complementary biomechanical tool that can visualize internal stress transfer and identify potential stress concentration sites within a construct under a prescribed load case (Phellan et al., 2021; Prendergast, 1997). Although suture-anchor fixation has been increasingly used for CC ligament reconstruction, most existing constructs remain based on independent PTP fixation, in which load imbalance between anchors may contribute to local stress concentration and anchor pullout. To our knowledge, the biomechanical behavior of a modified double-pulley configuration for CC ligament reconstruction has not been systematically evaluated. Therefore, this study aimed to compare the modified DP technique with the conventional PTP technique using synthetic bone biomechanical testing and FEA. The synthetic bone tests were used to quantify construct displacement under cyclic loading, ultimate load-to-failure, and failure modes, whereas FEA was used to compare relative load sharing and stress distribution between the two configurations under identical anatomical geometry and boundary conditions. We hypothesized that the modified DP technique would provide greater initial fixation strength, reduce cyclic displacement, and achieve more favorable stress distribution than the conventional PTP technique. Importantly, the FE model was not intended to predict in-vivo performance or absolute failure loads; rather, it provided a qualitative, mechanism-oriented comparison of the two fixation strategies under simplified loading conditions.

## 2 Materials and methods

### 2.1 Biomechanical testing

#### 2.1.1 Specimen preparation

A total of ten clavicle-coracoid polyurethane model pairs were prepared, with each pair consisting of two solid rigid polyurethane foam blocks (Block 20 PCF; 1522–03; Sawbone Europe AB, Malmö, Sweden) to simulate the distal clavicle and coracoid base. The ten model pairs were randomly assigned to the modified DP group or the PTP group, with five model pairs in each group. This type of polyurethane material has been validated in biomechanical pullout tests and is widely accepted as a reliable substitute for cancellous bone in mechanical testing models (Clevenger et al., 2014; Yakacki et al., 2009). The 20 PCF density was chosen to replicate normal cancellous bone properties (Clevenger et al., 2014). The dimensions of the foam blocks for the clavicle and coracoid were 60.0 mm × 20.0 mm × 15.0 mm and 60.0 mm × 20.0 mm × 16.0 mm, respectively (Rios et al., 2007; Schliemann et al., 2013).

Although the width and length of the synthetic clavicle and coracoid base models differ from human anatomical dimensions, a standardized insertion zone (14.0 mm wide and 23.0 mm long) was defined on the coracoid base to ensure anatomical relevance during anchor placement (Salzmann et al., 2008). The length and width of the clavicle and coracoid had minimal impact on the overall outcomes of this study. The choice of uniform block size was primarily to facilitate spatial alignment and temporary fixation of the reconstructed CC ligament (Figure 1A).

**FIGURE 1:**
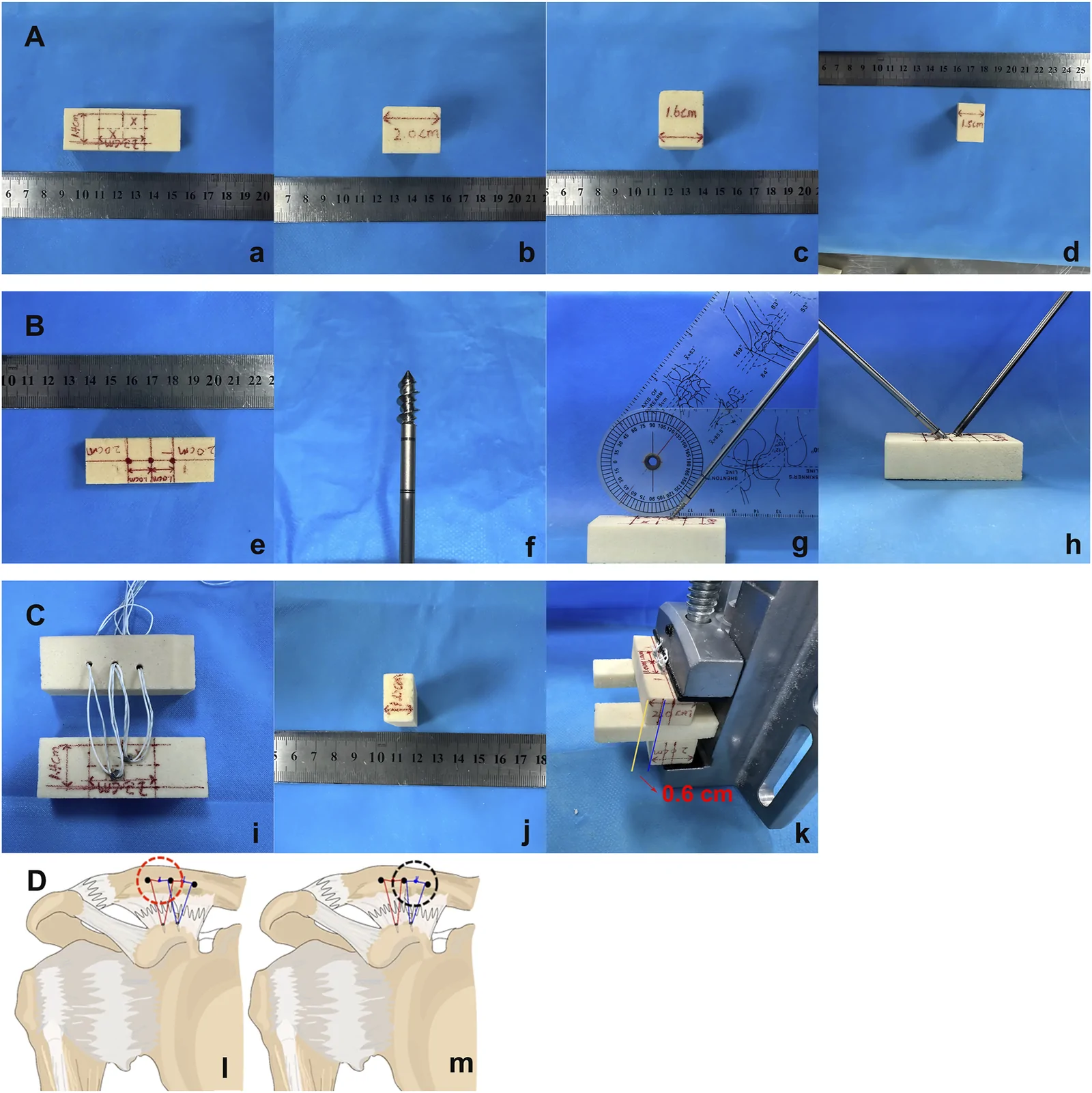
Polyurethane model preparation, tunnel construction, ligament reconstruction, and schematic diagrams of two surgical techniques. **(A)** Polyurethane foam bone models. Image a shows a model 6.0 cm in length, with a central area measuring 14.0 mm in width and 23.0 mm in length designated for anchor insertion. Image b shows the model width of 2.0 cm. Image c shows a simulated coracoid base thickness of 1.6 cm. Image d shows a simulated clavicle thickness of 1.5 cm. **(B)** Clavicle tunnel creation and anchor insertion. Image e shows a 6.0 cm clavicle model with three points selected at 1.0 cm intervals for tunnel creation. Image f shows a 5.0 mm suture anchor. Image g shows the anchor inserted at a 45° angle into the central region of the coracoid base. Image h shows the two anchors forming a 90° angle after insertion. **(C)** Ligament reconstruction model. Image i shows a 2/4/2 suture distribution passing through the clavicle tunnels. ImageJ shows a 1.0 cm-thick foam spacer used to simulate spatial construction. Image k shows the model fixed using a clamp, with the clavicle and coracoid offset by 6.0 mm. **(D)** Schematic diagrams of the modified double-pulley (DP) and point-to-point (PTP) techniques. Image l shows the modified DP technique: red and blue sutures tied within the red dashed circle. Image m shows the PTP technique: blue sutures tied within the black dashed circle.

#### 2.1.2 Ligament reconstruction techniques

Three evenly spaced points, 1.0 cm apart, were marked along the midline of the anterior-posterior axis on the clavicle model. At each marked point, a 2.0 mm Kirschner wire was used to drill bone tunnels from superior to inferior surfaces for suture passage. On the polyurethane coracoid model, two 5.0 mm double-loaded suture anchors were inserted at the central base region at a 45° angle in a crossed configuration (Figure 1B).

The eight suture limbs from the two anchors were passed through the three previously drilled clavicle tunnels in a 2/4/2 distribution. The clavicle and coracoid models were fixed using a custom jig, with a standardized alignment of 6.0 mm in the anterior-posterior direction, 10.0 mm in the superior-inferior direction, and a spatial distance of 12.0 mm between surfaces (Wellmann et al., 2007) (Figure 1C).

Two different reconstruction techniques were then performed by the same operator. In modified DP group, the modified DP technique was applied by interlacing and securing the sutures at the clavicle surface (Figure 1D). In PTP group, the PTP technique was performed, with direct fixation of the suture limbs at the clavicle without interconnection (Figure 1D).

#### 2.1.3 Biomechanical testing protocol

The reconstructed ligament model was first mounted onto a custom-designed fixture (Figure 2A) and secured to a mechanical tensile testing machine (Figure 2B). The cyclic loading protocol was adapted from previously published biomechanical studies of CC reconstruction that applied loads ranging from 20 to 70 N for 1,000 cycles as a standardized representation of repetitive loading during light early postoperative rehabilitation (Rios et al., 2007; Wellmann et al., 2007; Thomas et al., 2011; Voss et al., 2017). Each model first underwent 10 preconditioning cycles from 0 to 20 N at a crosshead speed of 1 mm/s to minimize initial settling effects. Cyclic loading was then applied vertically between 20 and 70 N at the same crosshead speed for 1,000 consecutive cycles. Initial displacement was defined as the peak displacement during the first loading cycle, whereas final displacement was defined as the peak displacement during the 1,000th cycle. Displacement change was calculated by subtracting the initial displacement from the final displacement. After cyclic loading, each model underwent load-to-failure testing at a crosshead speed of 50 mm/min, during which the ultimate load and failure mode were recorded. This protocol was intended to provide a standardized comparison between the reconstruction constructs rather than to reproduce the complete multidirectional loading environment of the shoulder.

**FIGURE 2:**
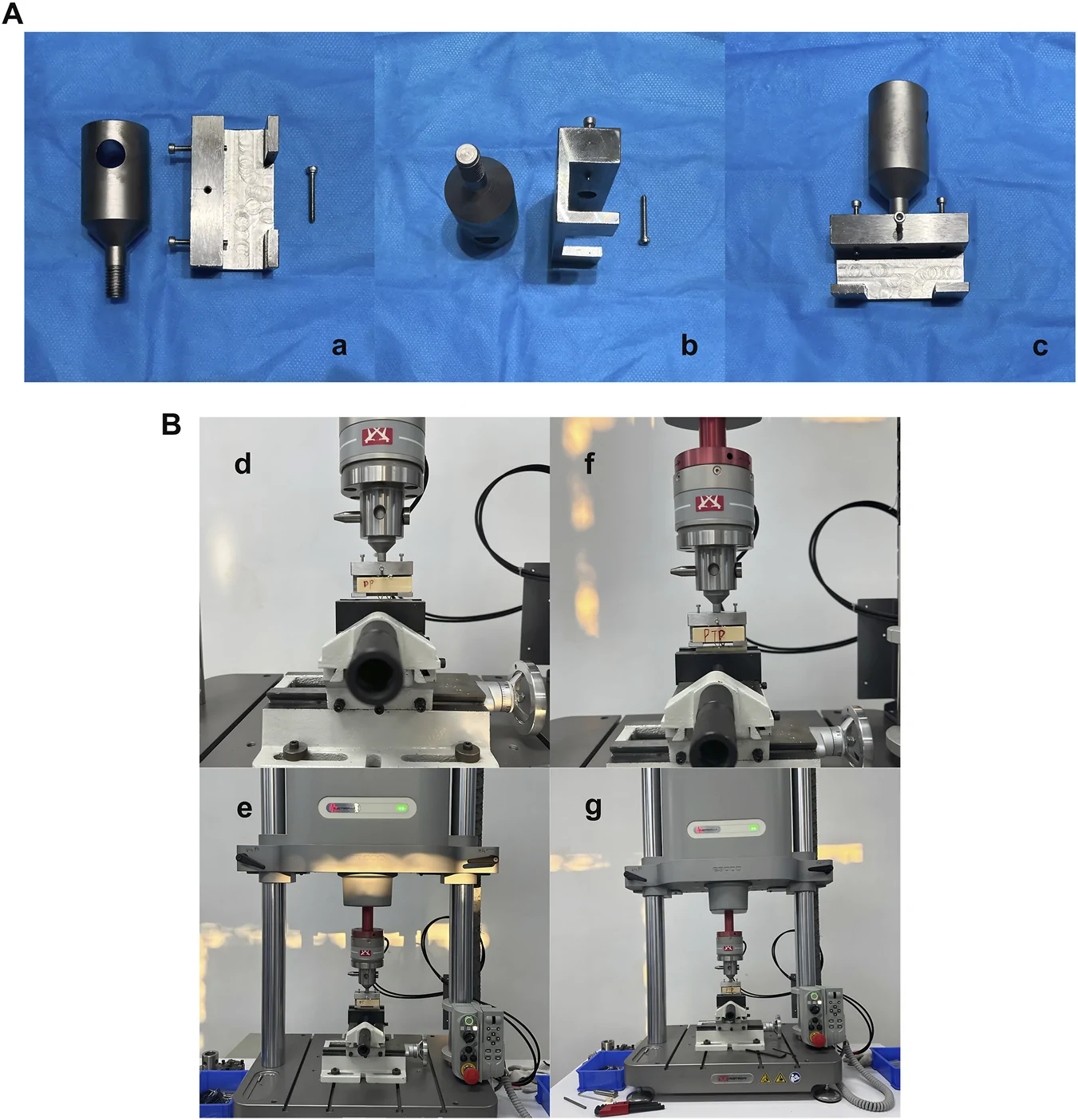
Mechanical testing setup and loading procedures for modified double-pulley (DP) and point-to-point (PTP) techniques. **(A)** Custom mechanical jig. Image a shows the front view of the disassembled components. Image b shows the side view of the disassembled components. Image c shows the front view of the assembled structure. **(B)** Mechanical loading of modified DP and PTP models. Images d and e show the loading setup for the modified DP technique. Images f and g show the loading setup for the PTP technique.

### 2.2 FEA

#### 2.2.1 Model construction

A healthy adult male volunteer (age: 32 years; height: 175 cm; weight: 68 kg) with no history of clavicle or scapular fractures, tumors, osteoporosis, or shoulder disorders was recruited for shoulder CT imaging after providing written informed consent. The use of the CT imaging data for finite element model construction was reviewed and approved by the Ethics Committee of The Affiliated Hospital of Southwest Medical University. CT scanning of the shoulder joint was performed using a GE LightSpeed VCT64 system in the Radiology Department, and the imaging data were saved in DICOM format.

The DICOM files were imported into Mimics 21.0 using the “New Project” module. Segmentation procedures, including thresholding, mask separation, mask editing, and cavity filling, were applied to extract the preliminary three-dimensional (3D) models of the clavicle and scapula (Figure 3A).

**FIGURE 3:**
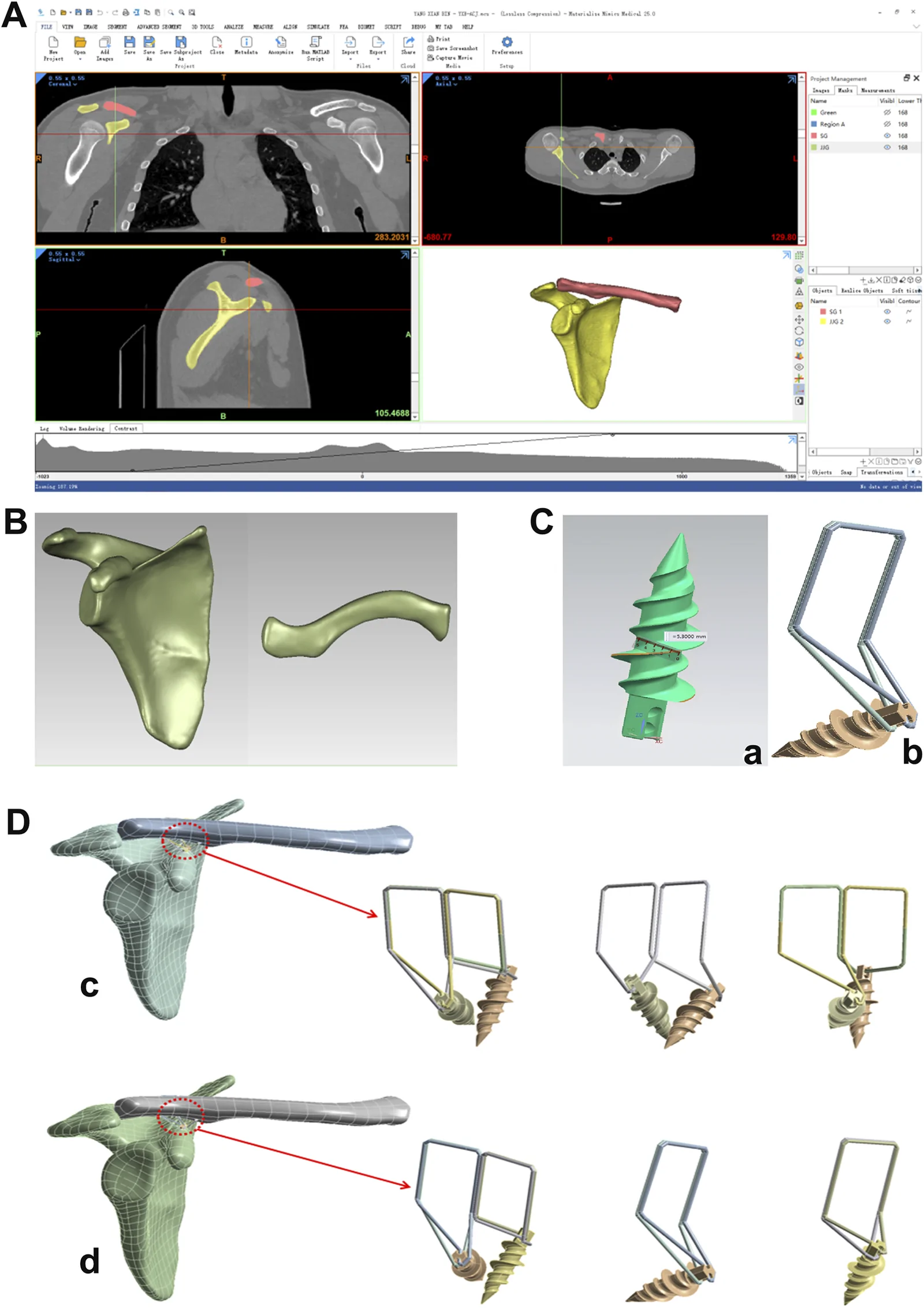
3D modeling and schematic diagrams of reconstruction techniques. **(A)** Clavicle and scapula models extracted using Mimics software. **(B)** Optimized models processed with Geomagic Wrap software. **(C)** Anchor models. Image a shows a 3D anchor model constructed in SolidWorks. Image b shows a suture anchor model combining the anchor with FiberWire. **(D)** Schematic diagrams of the modified DP and PTP reconstruction techniques. Image c represents the DP technique. Image d represents the PTP technique.

These 3D models were then imported into Geomagic Wrap software for noise reduction and surface smoothing to generate refined solid anatomical models (Figure 3B). The processed clavicle and scapula models, along with 3D-scanned suture anchor models, were assembled using SolidWorks software to construct the CC ligament reconstruction system (Figure 3C).

In both reconstruction models, two 5.0 mm suture anchors were implanted into the central base of the coracoid. Three 2.0 mm bone tunnels were drilled perpendicular to the midline at 2.5 cm, 3.5 cm, and 4.5 cm medial to the lateral end of the clavicle. Eight strands of No. Two FiberWire® sutures from the two anchors were passed through the tunnels in a “2/4/2” configuration. Model c represents the modified DP technique, and Model d represents the PTP technique (Figure 3D).

#### 2.2.2 Material properties and meshing

The finite element model consisted of four components: the clavicle, scapula (coracoid process), titanium alloy suture anchors, and No. Two FiberWire® sutures. All materials were assumed to be homogeneous, isotropic, and linearly elastic. Material properties were assigned based on values reported in previous studies (Lee et al., 2016; Menegaz et al., 2019). Two independent parameters, Young’s modulus (E) and Poisson’s ratio (ν), were used to characterize each material. For transparency and reproducibility, the exact E and ν values (with units) used in the FE model are provided in Table 1.

**TABLE 1:** Material parameters used in the model.

Component	Young’s modulus (MPa)	Poisson’s ratio (ν)
Scapula (coracoid)	16,000	0.3
Clavicle	11,000	0.3
Titanium suture anchor	118,000	0.3
Suture-FiberWire® no 2	10,729	0.4

Using FEA software, the shoulder reconstruction model was discretized into second-order tetrahedral elements (Figure 4). Mesh sizes were 5.0 mm for the clavicle and coracoid, 2.0 mm for the suture anchors, and 1.0 mm for the sutures. The FiberWire® sutures were modeled as three-dimensional solid elements to preserve the suture path and to allow surface contact definitions at the anchor eyelets and clavicular tunnels. Frictional contact at these regions was handled using the same contact pairs and coefficients described in Section 2.2.3. No separate edge-specific contact correction, local edge-radius sensitivity analysis, or solid-versus-cable/truss sensitivity analysis was performed. A tension-only cable or truss formulation was not selected because it would not preserve the three-dimensional contact path through the anchor eyelets and bone tunnels in the present comparative model. However, unlike a tension-only cable or truss formulation, a solid-element representation can carry compressive and bending stiffness that a real suture does not sustain, and may therefore alter local peak stresses and displacement magnitudes. The stresses around the anchor eyelets, tunnel apertures, and anchor-bone interface were therefore interpreted as qualitative relative results rather than absolute stress or failure predictions. The same component-specific target element sizes and mesh-control settings were applied to both reconstruction models to provide a consistent basis for comparison. However, only one mesh density was evaluated, and no formal mesh-convergence study based on sequential refinement and a predefined convergence criterion was performed. Mesh independence of the computed results was therefore not established. In particular, peak stresses around the bone tunnels, anchor eyelets, and anchor-bone interface may be sensitive to local element size, idealized edge geometry, and contact discretization. The element and node counts for both models are reported in Table 2.

**FIGURE 4:**
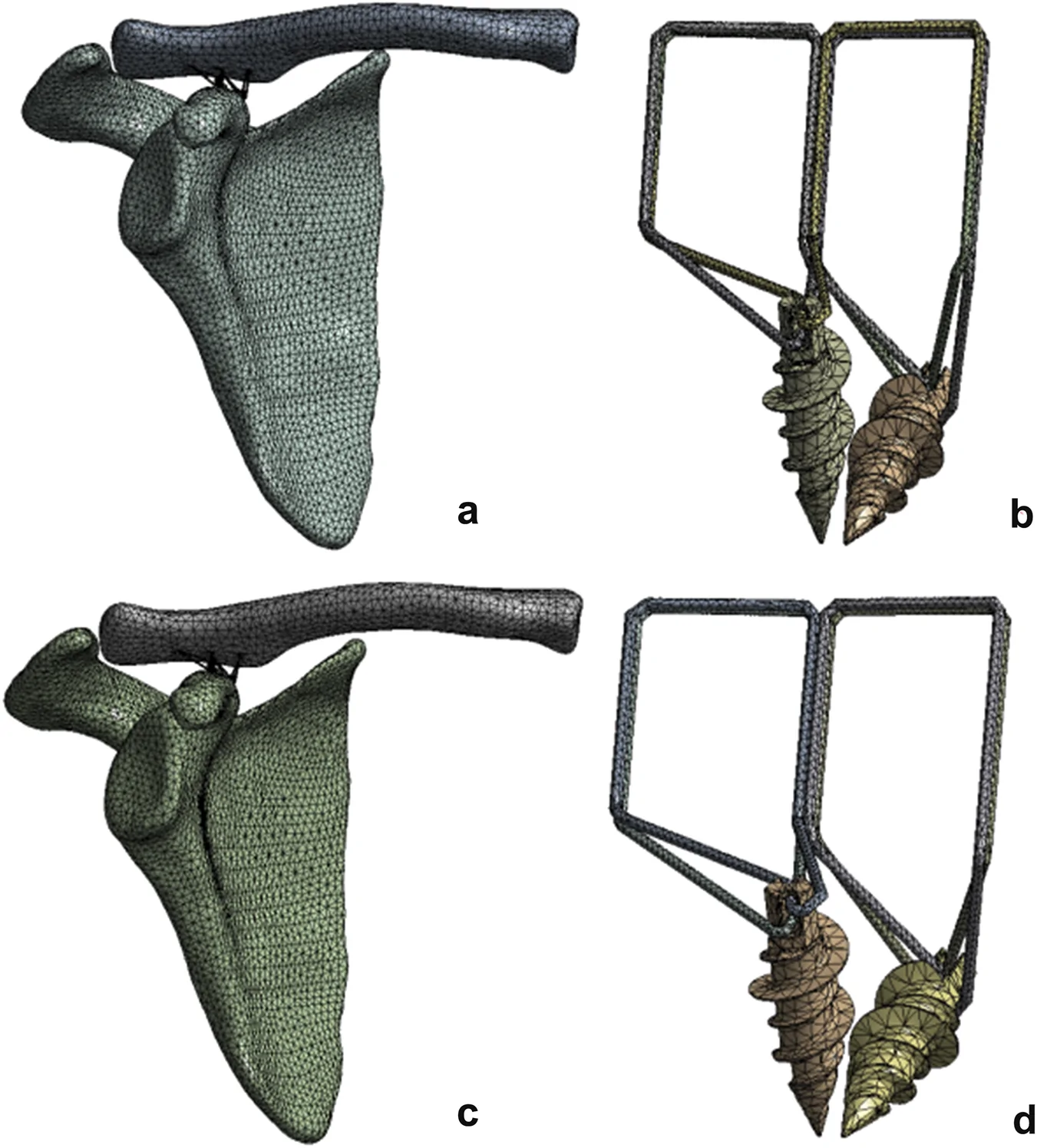
Mesh generation of finite element models. **(a,b)** Mesh distribution of the modified double-pulley (DP) reconstruction model. **(c,d)** Mesh distribution of the point-to-point (PTP) reconstruction model.

**TABLE 2:** Mesh characteristics of the finite element models.

Model	Number of nodes	Number of elements
Modified DP	132,804	79,110
PTP	131,076	78,141

#### 2.2.3 Boundary and loading conditions

After material assignment, loading and boundary conditions were established for a controlled comparative FEA. The coracoid/scapular base was fully constrained to prevent rigid-body motion. Frictional contact was defined between FiberWire® and the anchor (coefficient of friction, 0.31 (Wright et al., 2007)), between FiberWire® and the clavicular tunnels (0.46), and between the anchor and coracoid bone (0.40) (Hughes et al., 2014). The contact definitions covered the modeled surfaces surrounding the anchor-eyelet rims and tunnel apertures. No separate edge-specific friction law, edge-radius correction, or local contact sensitivity analysis was introduced. Peak stresses at these interfaces may therefore be sensitive to the local geometry and contact discretization and should not be taken as direct estimates of *in vivo* interface stress. A three-dimensional vector load was applied identically in both reconstruction models to simulate sternocleidomastoid traction on the clavicle when an adult raises a 0.5-kg object to approximately mouth level (Figure 5). The prescribed load components entered into the finite element model were $F_{x}=1.5\;N$ , $F_{y}=14.2\;N$ , and $F_{z}=-4.2\;N$ . The resultant magnitude of this prescribed vector load was calculated as $F=\sqrt{F_{x}^{2}+F_{y}^{2}+F_{z}^{2}}=\sqrt{1.5^{2}+14.2^{2}+{\left(-4.2\right)}^{2}}=14.884\;N$ . In Figure 5, this 14.884-N vector represents the applied external load. Force A (37.945 N) represents a calculated local reaction/mechanical response generated after the model reached equilibrium under the specified boundary constraints and internal force transmission; it was not prescribed as an additional external load. Identical loading and constraint conditions were applied to the modified DP and PTP models to permit comparison of relative load sharing and stress distribution. The load case did not incorporate anterior-posterior translation, rotational shear, multiple muscle forces, ligament constraints, or scapulothoracic motion; therefore, the FEA was used to compare the mechanical responses of the two reconstruction models rather than to predict their absolute *in vivo* stability or failure.

**FIGURE 5:**
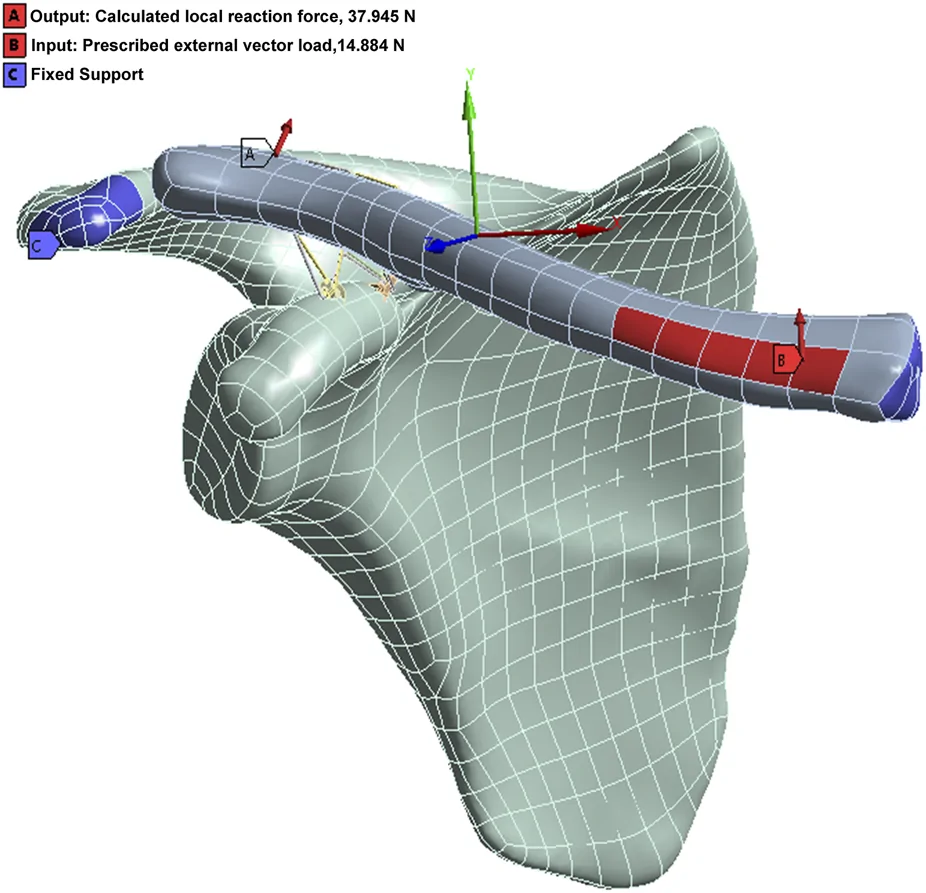
Boundary conditions and loading setup. The DP model is shown as an example. The coracoid/scapular base was fully constrained as a fixed support. The prescribed three-dimensional external vector load (Force B; input), used to represent the modeled sternocleidomastoid traction, consisted of $F_{x}=1.5\;N$ , $F_{y}=14.2\;N$ , and $F_{z}=-4.2\;N$ , corresponding to a resultant magnitude of 14.884 N. Force A (37.945 N; output) represents the calculated local reaction force generated once the model reached mechanical equilibrium under the prescribed external load, boundary constraints, and internal force transmission; it was not independently applied as an additional external load. Fixed-support regions are shown in blue.

### 2.3 Statistical analysis

Data were analyzed using SPSS 27.0 and are presented as mean ± standard deviation (SD). No *a priori* sample-size calculation was performed because this was an exploratory bench-top study. Normality was assessed using the Shapiro–Wilk test together with inspection of the individual data distributions. Because of the limited power of formal normality testing with five observations per group, the Shapiro–Wilk test was not used alone to justify parametric analysis. Two-sided independent-samples t-tests were used as the primary comparisons. To assess the robustness of the results without relying on the normality assumption, exact two-sided permutation tests were additionally performed by evaluating all 252 possible allocations of the 10 observations into two groups of five. Post-hoc achieved power was calculated descriptively from the observed pooled standardized mean difference, with α = 0.05 and five specimens per group; it was not treated as a substitute for an *a priori* calculation. A p-value <0.05 was considered statistically significant. In the figures, ** indicates p < 0.01 and *** indicates p < 0.001.

## 3 Results

### 3.1 Biomechanical testing

Under the 70 N cyclic loading condition, the peak displacement curves across 1000 cycles for modified DP group and PTP group are shown in Figure 6A. Modified DP group demonstrated a lower initial displacement peak than PTP group, indicating a smaller initial displacement. Modified DP group exhibited a lower final displacement peak and a smaller slope, suggesting less construct elongation during cyclic loading.

**FIGURE 6:**
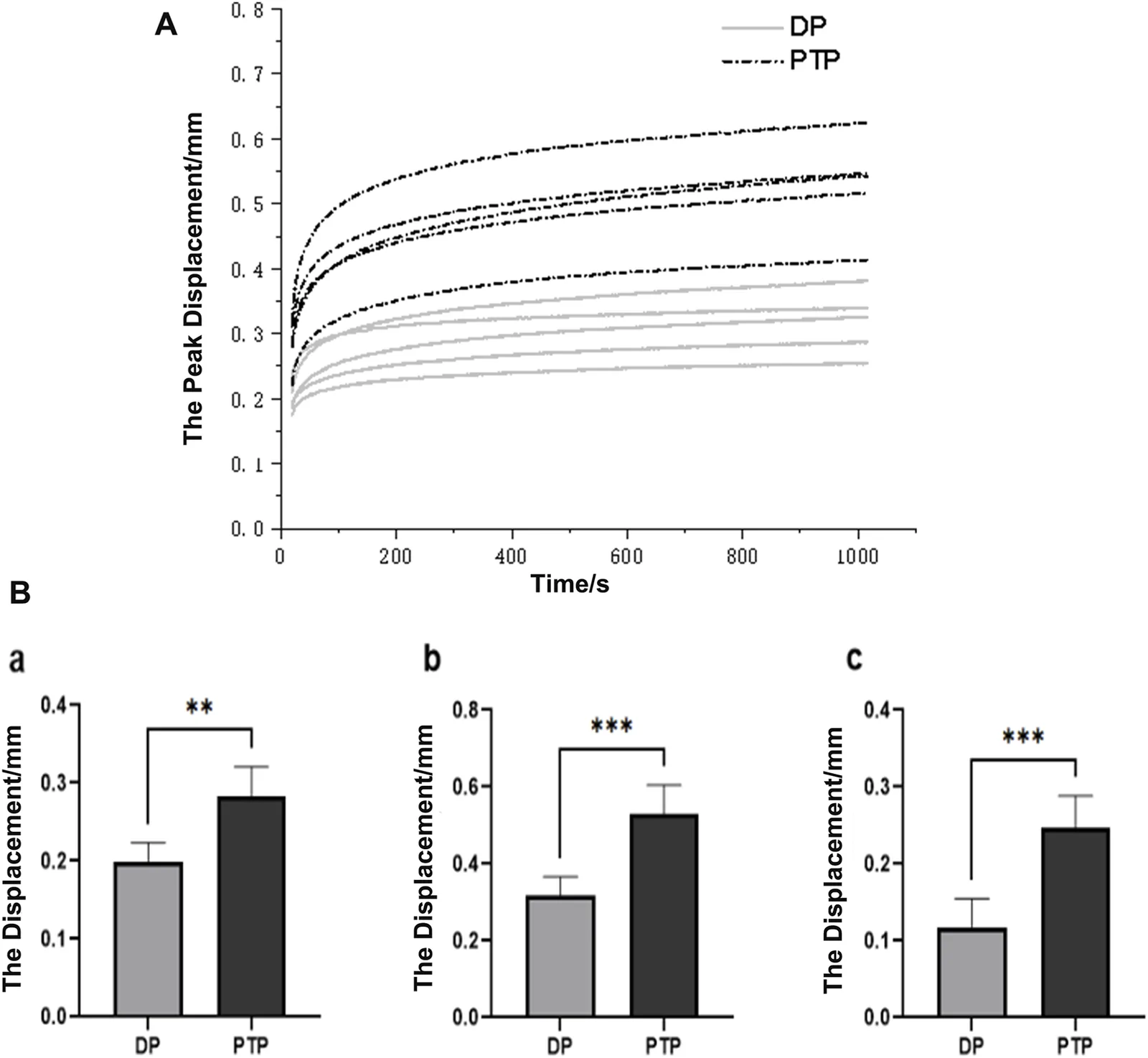
Cyclic loading test results. **(A)** Peak displacement curves during 1000 cycles of loading (solid line: modified DP group; dashed line: PTP group). **(B)** Independent-samples t-test comparisons of cyclic loading outcomes between groups (PTP group used as control). **P < 0.01, ***P < 0.001. **(a)** Initial peak displacement; **(b)** final peak displacement; **(c)** displacement change.

In modified DP group, initial displacement was 0.1997 ± 0.02417 mm, final displacement was 0.3174 ± 0.04878 mm, and displacement change was 0.1177 ± 0.03696 mm (Table 3). The corresponding values in PTP group were 0.2827 ± 0.03847 mm, 0.5288 ± 0.07621 mm, and 0.2461 ± 0.04248 mm, respectively (Table 4). All three outcomes were lower in modified DP group: initial displacement (p = 0.0035), final displacement (p = 0.0008), and displacement change (p = 0.0009) (Figure 6B). The corresponding post-hoc achieved powers were 94.5%, 99.5%, and 99.3%. Exact permutation testing confirmed the same direction and statistical significance (p = 0.0159, 0.0079, and 0.0079, respectively).

**TABLE 3:** The first and last displacements and the difference between the first and last displacements after 1000 cycles in modified DP group (mm).

Number	The first peak displacement in modified DP group (mm)	The last peak displacement in modified DP group (mm)	The difference between the first and the last displacement in modified DP group (mm)
**1**	0.2099	0.3811	0.1712
**2**	0.1865	0.3256	0.1391
**3**	0.2364	0.3390	0.1026
**4**	0.1913	0.2875	0.0963
**5**	0.1744	0.2539	0.0795
**Mean (** $\bar{X}$ **)**	0.1997	0.3174	0.1177
**Standard deviation (SD)**	0.02417	0.04878	0.03696

**TABLE 4:** The first and last displacements and the difference between the first and last displacements after 1000 cycles in PTP group (mm).

Number	The first peak displacement in PTP group (mm)	The last peak displacement in PTP group (mm)	The difference between the first and the last displacement in PTP group (mm)
**1**	0.2832	0.5164	0.2332
**2**	0.3123	0.5466	0.2344
**3**	0.2209	0.4133	0.1924
**4**	0.2796	0.5426	0.2630
**5**	0.3176	0.6249	0.3073
**Mean (** $\bar{X}$ **)**	0.2827	0.5288	0.2461
**Standard deviation (SD)**	0.03847	0.07621	0.04248

In the load-to-failure tests, all modified DP group specimens failed through pullout of both anchors, whereas all PTP group specimens failed through pullout of a single anchor (Figure 7A). The load-displacement curves are shown in Figure 7B. Mean ultimate load was higher in modified DP group than in PTP group (367.07 ± 17.244 N vs. 285.70 ± 38.482 N; p = 0.0026) (Figure 7C). The post-hoc achieved power for this comparison was 96.4%, and the exact permutation result remained significant (p = 0.0079).

**FIGURE 7:**
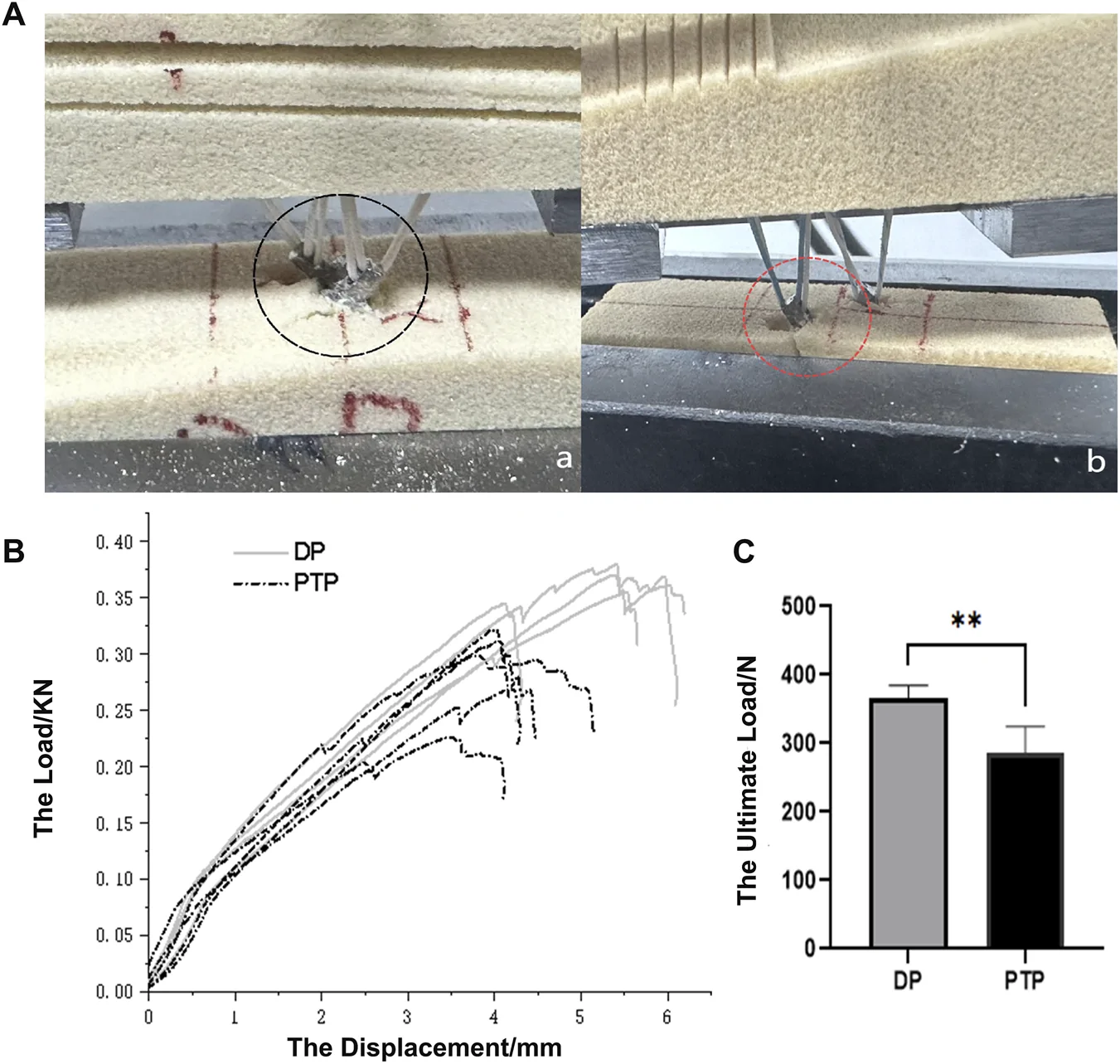
Load-to-failure test results. **(A)** Failure modes observed after tensile testing. Image **(a)** shows modified DP group with both anchors pulled out (black dashed circle); image **(b)** shows PTP group with a single anchor pulled out (red dashed circle). **(B)** Load-displacement curves during tensile testing (solid line: modified DP group; dashed line: PTP group). **(C)** Independent-samples t-test comparison of ultimate load, with the PTP group as control. **P < 0.01.

### 3.2 FEA

Under identical boundary and loading conditions, the maximum displacement occurred at the distal end of the clavicle in both reconstruction models. In both groups, the lateral suture anchors and their tails experienced higher stress compared to the medial ones. However, the modified DP technique allowed partial stress transfer to the medial anchor, while the PTP technique resulted in greater stress concentration on the lateral anchor.

Under the specified mesh and modeling conditions, the computed peak suture stress was 287.72 MPa in the PTP model and 196.01 MPa in the modified DP model; the value in the modified DP model was 31.87% lower. Similarly, the computed peak anchor stress occurred at the lateral anchor in both models and was 274.82 MPa in the PTP model and 196.01 MPa in the modified DP model, corresponding to a model-specific difference of 28.68% (Figures 8A,B). Under the present simplified loading and solid-element modeling conditions, the DP model showed lower computed peak stress at the lateral anchor-suture region than the PTP model.

**FIGURE 8:**
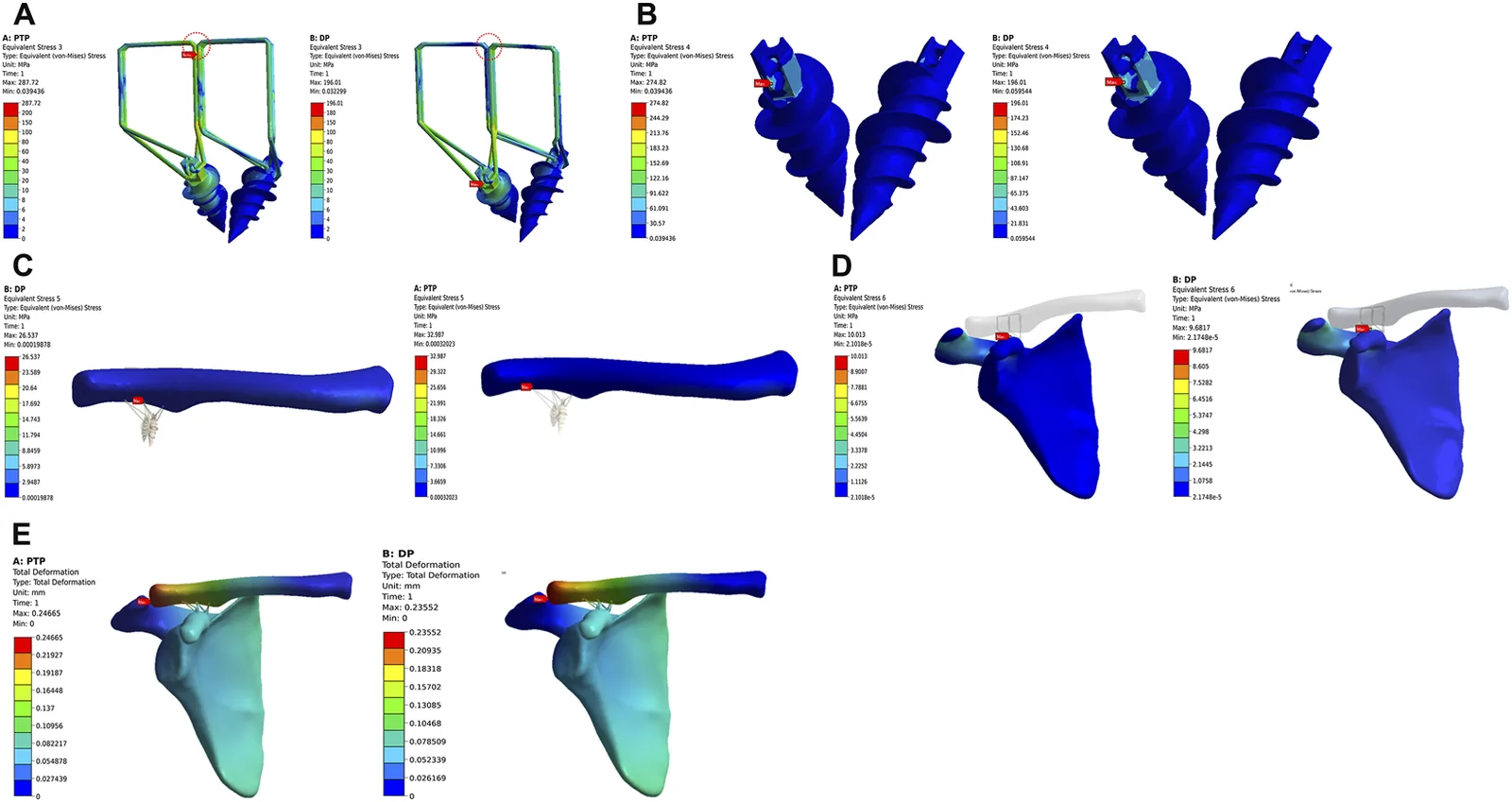
FEA of stress and displacement distributions. **(A)** Suture stress distribution maps. The maximum suture stress was 287.72 MPa in the PTP model and 196.01 MPa in the modified DP model. **(B)** Anchor stress distribution maps. The maximum anchor stress was 274.82 MPa in the PTP model and 196.01 MPa in the modified DP model. **(C)** Clavicle stress maps. The modified DP model exhibited a peak stress of 26.537 MPa, located at the central tunnel, while the PTP model exhibited a peak stress of 32.987 MPa at the distal tunnel. **(D)** Scapula/coracoid stress maps. The PTP model showed a peak stress of 10.013 MPa, whereas the modified DP model showed a slightly lower peak stress of 9.6817 MPa. **(E)** Total displacement maps. The PTP model showed a distal clavicle displacement of 0.24665 mm, while the modified DP model showed a smaller displacement of 0.23552 mm. Within each panel, the PTP and DP models are shown according to the labels in the figure.

Regarding clavicle stress (Figure 8C), the modified DP model demonstrated lower peak stress (26.537 MPa) than the PTP model (32.987 MPa). The maximum stress occurred at the central tunnel in the modified DP model and at the distal tunnel in the PTP model. For the scapula (Figure 8D), the PTP model exhibited a peak stress of 10.013 MPa, while the DP model showed a slightly lower value of 9.6817 MPa. As for distal clavicle displacement (Figure 8E), the PTP model reached 0.24665 mm, whereas the DP model achieved 0.23552 mm. Overall, the modified DP model showed lower computed peak stresses in the suture, anchor, clavicle, and scapula/coracoid components, together with a small reduction in distal clavicle displacement, under the same simplified loading and modeling conditions.

## 4 Discussion

AC joint dislocations remain a challenging injury in orthopedics, and the choice of fixation method directly affects postoperative stability and long-term outcomes. In this study, we compared a modified DP technique with a conventional PTP technique for CC ligament reconstruction using synthetic bone biomechanical testing and FEA. The principal finding was that the modified DP construct showed lower displacement during cyclic loading and a higher ultimate failure load than the PTP construct. In addition, failure in the modified DP group occurred through bilateral anchor pullout, whereas the PTP group failed through unilateral anchor pullout, suggesting a more balanced load-sharing pattern in the DP configuration. The FEA provided qualitative and model-dependent support for this mechanical interpretation by showing lower computed peak stresses in the sutures, anchors, clavicle, and scapula/coracoid region under identical simplified loading conditions. Because the sutures were represented by three-dimensional solid elements and no tension-only sensitivity analysis was performed, these findings should not be interpreted as formulation-independent evidence of superior load sharing. These findings indicate that the modified DP technique may improve initial construct stability and reduce local stress concentration compared with the conventional PTP method. However, because this study was based on synthetic bone models and simplified FEA, the results should be interpreted as preliminary biomechanical evidence rather than direct proof of improved clinical outcomes.

Proper positioning of clavicular bone tunnels is essential for restoring CC ligament function, as malposition may compromise postoperative stability and increase the risk of redislocation. Previous anatomical studies in Chinese populations reported that the conoid and trapezoid ligament footprints are located approximately 25% and 16% of the clavicular length from the lateral end, respectively (Xue et al., 2013; Eisenstein et al., 2016). These findings support the clinical practice of placing tunnels at about 2.5–4.5 cm from the distal clavicle. Consistent with these anatomical benchmarks, we adopted a tri-tunnel configuration at 2.5, 3.5, and 4.5 cm with 1.0 cm spacing. This design not only aligns with cadaveric reconstructions but also provides favorable stress distribution for the modified DP technique while minimizing bone weakening and surgical trauma (Liu et al., 2020; Rios et al., 2007; Carofino and Mazzocca, 2010).

The AC joint exhibits complex kinematics involving rotation, adduction, abduction, and pendulum-like motion. While the AC ligaments and joint capsule primarily restrain horizontal translation, vertical stability is largely governed by the CC ligaments, comprising the trapezoid and conoid bundles, each playing distinct roles in joint stabilization (Oki et al., 2012). Cadaveric studies have shown that among the CC ligament bundles, the conoid ligament plays the dominant role in maintaining vertical stability of the AC joint (Oki et al., 2012). Biomechanical studies indicate that the conoid ligament is the primary vertical load-bearing structure of the CC complex, while the trapezoid provides secondary stability, mainly resisting posterior displacement, and exhibits more uniform strain across a wider abduction range, suggesting that anatomical reconstruction should prioritize conoid tunnel placement (Salter et al., 1987; Fukuda et al., 1986; Lee et al., 1997; Izadpanah et al., 2012). The trapezoid ligament may be fixed at any abduction angle within 0°–120°. In this study, two suture anchors were used to mimic the dual-bundle nature of the CC ligament. FEA revealed that the lateral anchor, simulating the trapezoid component, bore higher stress levels, which is consistent with clinical observations that trapezoid disruption often precedes conoid injury during progressive trauma to the AC joint.

The AC joint is a diarthrodial articulation that supports upper limb suspension and force transmission during complex shoulder movements. High-grade dislocations of this joint often alter scapular kinematics, leading to functional impairment (Oki et al., 2012). As anatomical and biomechanical understanding of this joint deepens, researchers such as Debski et al. have characterized the AC joint as a structure stabilized by the coordinated function of multiple ligaments, rather than a single linear constraint (Debski et al., 2000). Surgical intervention is generally recommended for Rockwood type IV-VI injuries and select type IIIB cases to restore anatomic alignment and facilitate ligamentous healing (Beitzel et al., 2014). In CC ligament reconstruction using suture anchors, the choice between one versus two anchors significantly affects biomechanical outcomes. A single anchor may be prone to failure under unidirectional tensile load due to stress concentration at the bone-anchor interface. In contrast, dual-anchor constructs distribute load more evenly and better replicate the native dual-bundle anatomy of the conoid and trapezoid ligaments (Liu et al., 2020). The DP technique, initially described by Arrigoni et al. for rotator cuff repair (Arrigoni et al., 2007), utilizes crossing sutures to convert linear traction into a downward compressive force, enhancing tissue apposition and increasing resistance to pullout. Although successfully applied in treating rotator cuff tears, scapular glenoid fractures, acetabular rim fractures, and patellar avulsions (Fang et al., 2023; Guo and Jiang, 2021; Wang et al., 2022; Yin et al., 2022), its use in AC joint reconstruction has not been previously reported. In this study, polyurethane foam blocks were used to replicate cancellous bone, and anchor positioning followed validated anatomic and mechanical standards (Rios et al., 2007). Spacing between the clavicle and coracoid was fixed at 12 mm (anteroposterior: 6 mm, superoinferior: 10 mm) to simulate physiologic alignment.

Biomechanical testing confirmed that the modified DP group exhibited lower initial and final displacement under cyclic loading than the PTP group, indicating enhanced early fixation strength under the present test conditions. Moreover, the average ultimate load in the DP group was significantly higher (367.07 ± 17.24 N vs. 285.70 ± 38.48 N), with all failures occurring through anchor pullout. Notably, DP constructs consistently failed via bilateral pullout, suggesting balanced force distribution, while PTP failures were unilaterally concentrated. The FEA results provided qualitative, model-dependent mechanistic support rather than independent validation of clinical failure behavior. In the present solid-element model, the modified DP configuration showed lower computed peak stress values in the suture (196.01 MPa vs. 287.72 MPa), anchors (196.01 MPa vs. 274.82 MPa), and clavicle (26.54 MPa vs. 32.99 MPa) compared to the PTP configuration, corresponding to model-specific reductions of 31.87%, 28.68%, and 19.55%, respectively. These differences may be related to the looped configuration of the DP construct, which creates a mechanically coupled system capable of distributing tensile forces more efficiently (Lee et al., 2016; Wright et al., 2007). Similar effects of reconstruction geometry on load transfer and stress distribution have been described in previous biomechanical and finite element studies of coracoclavicular reconstruction (Clevenger et al., 2014; Schliemann et al., 2013; Menegaz et al., 2019). Because the DP and PTP constructs differed in suture path, contact angle, and local bending configuration, the solid-element formulation may have affected the two models unequally. The reported stress differences should therefore be regarded as exploratory, model-dependent findings rather than formulation-independent estimates of stress reduction or direct predictions of construct failure. A 2.0-mm tunnel diameter was used in the present model. Although smaller tunnels may limit tunnel-related bone compromise (Hughes et al., 2014), the risk of iatrogenic fracture, particularly in osteoporotic bone, was not directly evaluated in this study. The cyclic displacement curves showed that most construct elongation occurred during the first 200 cycles, after which the displacement approached a plateau. This pattern may reflect initial settling or suture tightening under the present *in vitro* loading conditions. Whether this behavior corresponds to early postoperative relaxation *in vivo*, or should influence any specific intraoperative reduction strategy, requires further investigation.

The bilateral anchor pullout observed in the modified DP construct may have clinically relevant implications. Because the two anchors form an interconnected load-sharing loop, loss of fixation at one anchor may redistribute load abruptly to the remaining fixation point and disrupt the entire construct, potentially resulting in complete loss of reduction. In contrast, the relatively independent fixation paths of the PTP construct may allow unilateral anchor failure to occur first while the contralateral fixation point temporarily preserves partial stability, producing a more sequential failure pattern. Nevertheless, this interpretation is mechanistic and should not be taken as evidence that the modified DP technique has a higher clinical incidence of catastrophic failure, because residual stability after isolated anchor failure and clinical failure rates were not evaluated. When the modified DP technique is used, particularly in patients with compromised bone quality, careful evaluation of bone stock, adequate anchor spacing and trajectory, avoidance of cortical breach, and balanced tensioning are important. An independent backup loop, cortical-button augmentation, tendon graft, or other hybrid fixation may also be considered when anchor purchase is uncertain.

This study has several limitations. First, the 20 PCF polyurethane blocks standardized cancellous bone density but lacked a cortical shell and could not reproduce the cortical-cancellous interface, anatomical geometry, or biological variability of the human clavicle and coracoid. Consequently, the measured pullout loads should not be extrapolated directly to elderly or osteoporotic bone, in which anchor purchase would be expected to be less reliable. Second, only five specimens were tested per group and no *a priori* power analysis was performed. Although the observed effect sizes yielded post-hoc powers of 94.5%–99.5% and the exact permutation analyses supported the primary comparisons, these results do not remove the uncertainty associated with a small exploratory sample. Third, the mechanical tests used a narrow uniaxial 20–70 N loading protocol, and the finite element model used a low prescribed vector load. Therefore, the present study cannot determine whether the modified DP construct would maintain the same structural advantage under higher repetitive loads, such as 100–300 N cyclic ranges, or under combined vertical, horizontal, and rotational loading. Although the double-pulley configuration may theoretically improve load sharing between the two fixation points, repetitive changes in loading direction and angle may induce suture binding, abrasion, local shear, or cut-through. These potential failure mechanisms, together with anterior-posterior translation, rotational shear, and scapulothoracic motion, could not be evaluated using the present simplified loading protocol and FEA model. Future studies may extend the present controlled comparison by incorporating multiaxial cyclic loading, anterior-posterior translation, rotational shear, and direct assessment of suture abrasion, binding, and construct failure modes. Fourth, the FEA was based on a single healthy 32-year-old male and therefore did not capture variation in clavicular and coracoid geometry or bone density across patients. The prescribed load values were not derived from a validated physiological loading model. In addition, the use of homogeneous, isotropic, linearly elastic materials and three-dimensional solid suture elements may have artificially introduced compressive and bending stiffness and altered local load transfer, stress concentrations, and displacement predictions. Because the modified DP and PTP constructs differed in suture path, contact angle, and local bending configuration, this modeling bias may not have affected the two constructs equally. No formal mesh convergence analysis was performed, and the local peak stresses around the bone tunnels, anchor eyelets, and anchor-bone interface may therefore be sensitive to element size, local geometry, and contact definitions. Accordingly, the absolute FEA values cannot be considered mesh-independent, and the observed between-construct differences remain exploratory and specific to the present discretization and modeling assumptions. Because no sensitivity analysis comparing solid elements with tension-only cable or truss formulations was performed, the magnitude and direction of this effect could not be quantified. Future studies should assess whether the principal findings remain consistent under tension-only suture formulations and refined meshes. Suture pretension, knot slippage, soft-tissue healing, and bone remodeling were also not simulated. Finally, no fresh-frozen cadaveric validation or clinical follow-up was performed. Further work may examine these findings in anatomically variable and osteoporotic models and assess their robustness using refined meshes, alternative suture formulations, multiaxial loading, and complementary clinical follow-up.

## 5 Conclusion

The modified DP technique demonstrated greater initial fixation strength and reduced cyclic displacement compared with the conventional PTP method under the present synthetic-model testing conditions. Under the specified finite element mesh and modeling assumptions, the modified DP model also showed lower computed peak stresses and distal clavicular displacement. These finite element findings are exploratory and model-dependent and should not be interpreted as mesh-independent estimates or direct predictions of clinical performance. Collectively, the results support further biomechanical and clinical evaluation of the modified DP configuration.

## Data Availability

The original contributions presented in the study are included in the article/Supplementary Material, further inquiries can be directed to the corresponding authors.
